# Laparo-Endoscopic Single-Site Surgery for Radical and Cytoreductive Nephrectomy, Renal Vein Thrombectomy, and Partial Nephrectomy: A Prospective Pilot Evaluation

**DOI:** 10.1155/2010/107482

**Published:** 2010-06-07

**Authors:** Ithaar H. Derweesh, Jonathan L. Silberstein, Wassim Bazzi, Ryan Kopp, Tracy M. Downs, Christopher J. Kane

**Affiliations:** ^1^Division of Urology, Department of Surgery, University of California, San Diego School of Medicine, San Diego, CA 92103, USA; ^2^Moores UCSD Cancer Center, University of California, San Diego School of Medicine, San Diego, CA 92093, USA

## Abstract

*Introduction*. Laparo-endoscopic single-site surgery (LESS) may diminish morbidity of laparoscopic surgery. We prospectively evaluated feasibility and outcomes of LESS-Radical Nephrectomy (LESS-RN) and Partial Nephrectomy (LESS-PN). *Methods*. 10 patients underwent LESS-RN (6) and LESS-PN (4) between 2/2009-5/2009. LESS-RN included 2 with renal vein thrombectomy, one of which was also cytoreductive. Transperitoneal LESS access was obtained by periumbilical incision. Patient/tumor characteristics, oncologic, and quality of life (QoL) outcomes were analyzed. *Results*. 3 Men/7 Women (mean age 58.7 years, median follow-up 9.8 months) underwent LESS. 9/10 cases were completed successfully. All had negative margins. Mean operative time was 161 minutes, estimated blood loss was 125 mL, and incision size was 4.4 cm. Median tumor size for LESS-RN and -PN was 5.0 and 1.7 cm (*P* = .045). Median LESS-PN ischemia time was 24 minutes; mean preoperative/postoperative creatinine were 0.7/0.8 mg/dL (*P* = .19). Mean pain score at discharge was 1.3. Mean preoperative, 3-, and 6-month postoperative SF-36 QoL Score was 73.8, 74.4 and 77.1 (*P* = .222). All patients are currently alive. *Conclusions*. LESS-RN, renal vein thrombectomy, and PN are technically feasible and safe while maintaining adherence to oncologic principles, with excellent QoL preservation and low discharge pain scores. Further study is requisite.

## 1. Introduction

Incidence of Renal Cell Carcinoma is increasing world wide. In 2009, approximately 57,760 patients were diagnosed with kidney cancer in the United States alone [1, 2]. Surgical excision remains the mainstay of therapy for localized disease and a cornerstone of care form appropriate patients with advanced disease. Since introduction of laparoscopic radical nephrectomy (LRN) the procedure has been refined and adopted as a standard of care for appropriate renal masses, with advantages including decreased blood loss, lower narcotic requirements, shorter hospital stays, and more rapid return to normal activities while maintaining equivalent short- and long-term oncologic efficacy compared with open radical nephrectomy [3–5]. These improved outcomes are thought in large part to be a result of the smaller incisions, and thus laparoscopy has rapidly emerged as standard of care for radical nephrectomy [6]. Similar to the introduction of LRN with its equivalent oncologic outcomes and improved morbidity profile, with the advent of improved laparoscopic surgical instrumentation and refinements in technique, LPN has emerged as a viable alternative to open partial nephrectomy (OPN) with equivalent short- and intermediate-term outcomes for selected patients [7, 8]. 

By combining working trocar sites and the eventual extraction site into a single location, Laparo-endoscopic single-site surgery (LESS) further limits the invasiveness of laparoscopy and may enhance advantages associated with traditional laparoscopy. Reduced incisional morbidity and improved cosmesis have largely sparked a growing interest in utilization of this technique to perform upper tract urologic surgery [9–11]. Concerns regarding the applicability of LESS are significant and center around the issues of restricted freedom of movement resulting in instrument clashing and lack of triangulation [9–11]. Herein we describe our technique and our initial prospective evaluation of LESS Radical Nephrectomy (LESS-RN) and Partial Nephrectomy (LESS-PN) for excision of enhancing renal masses and evaluate its short-term disease-based and quality of life impact.

## 2. Methods

### 2.1. Patient Selection

**Figure 1:**
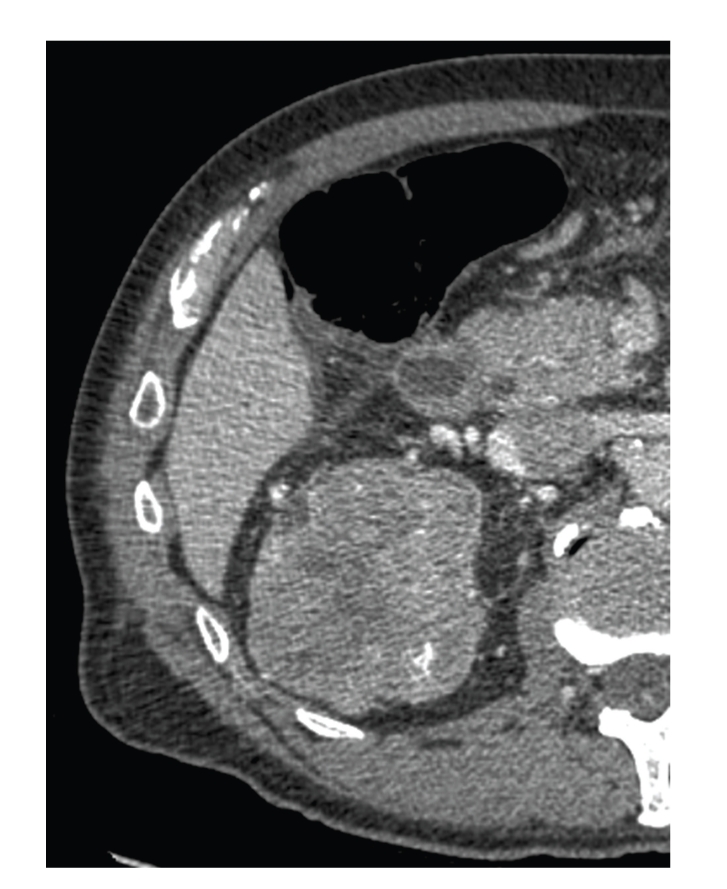
Representative image of a large (7 × 8 cm right upper pole mass) which underwent LESS-RN.

Prospective single institutional evaluation of LESS is performed by a single surgeon (IHD). Patients were referred with renal masses with radiographic criteria for suspicious for malignancy (Figure 1). All patients underwent complete history and physical exam and staging workup (chest/abdominal/pelvic computed tomography, liver function tests, and bone scintigraphy if necessary). Exclusion criteria for LESS included patients with imperative criteria for partial nephrectomy (solitary kidney, bilateral tumors, and preexisting nondialysis-dependent renal insufficiency), and tumors which crossed the midline, or those with bulky lymphadenopathy. Patients are considered for LESS-PN if they had a renal mass that was deemed amenable to laparoscopic partial nephrectomy (LPN). Patients were considered for LESS-RN if they were not deemed to be amenable to LPN and were candidates for elective OPN, but stated an explicit preference for RN despite potential feasibility of OPN. All procedures were consecutively performed between 2/2009 and 6/2009. Patient demographic factors, tumor characteristics, perioperative variables, outcomes and complications, and quality of life (QoL) scores were recorded at time of enrollment and analyzed.

### 2.2. Surgical Technique for LESS-RN

**Figure 2:**
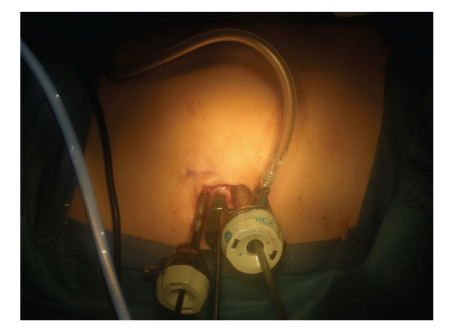
LESS Platform, periumbilical incision for left radical nephrectomy, demonstrating, in cranial to caudal direction (left to right): 5 mm Extralong Xcel Trocar, 5 mm short nonshielded trocar, and 12 mm Xcel Trocar.

After general anesthesia, the patient is placed in a modified flank position (with the patient at a 30 degree angle with the kidney rest up and the table flexed). A 3-4 cm periumbilical incision is made and carried down to the anterior abdominal wall fascia. A 5 mm extralong (150 mm length) Xcel trocar (Ethicon-Endosurgery, Cincinnati, OH) is then inserted at the most cranial aspect of this incision, at the junction of the umbilicus with the fascia; pneumoperitoneum to 15 mmHg is obtained and a 5 mm-zero degree 35 cm-long laparoscope (Strkyer, Kalamazoo, MI) is inserted to inspect the abdomen; subsequently a 65 mm-long, nonshielded low profile (65 mm length) trocar (Ethicon) is inserted under direct vision at a position 1.0–1.5 cm caudad to the initial port, followed by insertion of a standard length (100 mm) 12 mm Xcel trocar (Ethicon) at the most caudal aspect of the incision, another 1.0–1.5 cm caudad to the prior low profile port. We minimized the intracorporeal profile of the Xcel trocars, and that in conjunction with the variety of trocar lengths allowed us to stagger the external profiles in order to minimize instrument clashing (Figure 2). 

Tissue dissection is largely performed with standard extralong laparoscopic instruments (nonlocking laparoscopic deBakey bowel forceps, right angle dissector, Maryland dissector, endoshears) and 5 mm harmonic ACE 36 cm curved shears (Ethicon). Utilization of extralong instruments creates extracorporeal triangulation which compensates for the intracorporeal triangulation afforded by spaced trocars in multisite laparoscopy. Following takedown of the white line of Toldt, the 0 degree laparoscope is exchanged for a 5 mm, 45 cm, and 30 degree laparoscope with a right angle adaptor and inline camera head (Strkyer). 

**Figure 3:**
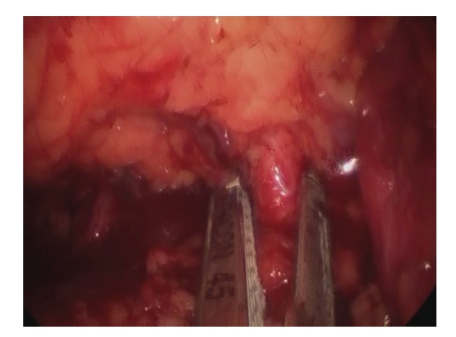
Ligation of right renal vein with the 45 mm Endopath ETS Flex Articulating Linear Cutter.

LESS-RN recapitulates standard LRN technique [3]. This includes incision of the descending colonic attachments to the abdominal sidewall along the white line of Toldt down to the level of the iliac vessels with subsequent medial-visceral rotation of the colon to expose the ureter and underling kidney. The ureter is identified, clipped, and cut using 5 mm Ligamax Clip applier (Ethicon) and endoshears. The cut proximal ureter is then used for retraction allowing safe access to the renal hilum, and the renal artery and vein are individually dissected. Through the 12 mm port the Endopath ETS Flex 45 Endoscopic Articulating Linear Cutter, with a white vascular reload (Ethicon), is used to ligate and incise the renal artery followed by the renal vein (Figure 3). In the case of an upper pole or locally advanced mass, the adrenal gland is then taken en bloc with the kidney and the remaining superior and lateral renal attachments are freed utilizing the harmonic scalpel, and in the case of a lower- or mid-pole mass, the adrenal gland is dropped away from the kidney (after ligation on the main adrenal vein on the left with the stapler and/or harmonic scalpel; or on the right after separation from the kidney utilizing the harmonic scalpel or Endovascular stapler). The 12 mm trocar is then exchanged for a 15 mm Xcel bladeless trocar and the specimen is placed in a 15 mm Endo Catch specimen pouch (Covidien, Mansfield, MA) and intact specimen extraction is made by extending the fascial opening between trocars.

### 2.3. Surgical Technique for LESS-PN with Ischemic Technique

Initial surgical steps including positioning, single-site incision, pneumoperitoneum/trocar placement, colonic mobilization, and ureteral identification and vascular dissection are identical to LESS-RN. The standard techniques of LPN are recapitulated with few modifications [12]. Following hilar control and tumor identification, Gerota's fascia is incised down to the renal capsule, preserving fat overlying the tumor. Subsequently, argon beam coagulator (ABC, Conmed, Centennial, CO) is used to circumscribe the renal capsule while leaving a healthy margin around the tumor. Renal hilar occlusion is obtained by placing laparoscopic vascular bulldog clamps (Aesculap, Center Valley, PA) on the renal artery and vein. Cold shears are then used to excise the lesion, making certain to leave a margin of healthy tissue around the specimen. Hemostasis is then achieved, first using ABC over the resected tumor bed. Next floseal (Baxter, Deerfield, IL) is placed in renal defect, followed by a surgicel bolster (Ethicon, Somerville NJ) and then sutured renorrhaphy [13] with 3-0 vicryl sutures on an SH needle are precut 10 cm long with a Lapra-Ty clip (Ethicon) on the free end. Simple interrupted sutures are placed in the renal defect with second Lapra-Ty clip on the distal end. Bioglue (albumin-glutaraldehyde sealant-adhesive, Cryolife, Kennesaw, GA) is then placed to seal the renorrhaphy [14] and then the laparoscopic bulldogs are removed, and the renorrhaphy is inspected for bleeding. The specimen is then extracted through a 5 mm Endo Catch specimen pouch in a similar fashion to the LESS-RN. 

### 2.4. Surgical Technique for Nonischemic LESS-PN

In one case, the Habib 4X (Angiodynamics, Queensbury, NY), a bipolar laparoscopic radiofrequency ablation device, was utilized to achieve a zone of parenchymal hemostasis in the absence of ischemic renal conditions prior to cold tumor excision and renorrhaphy, as described above [15, 16]. 

### 2.5. Postoperative Management and Followup

**Figure 4:**
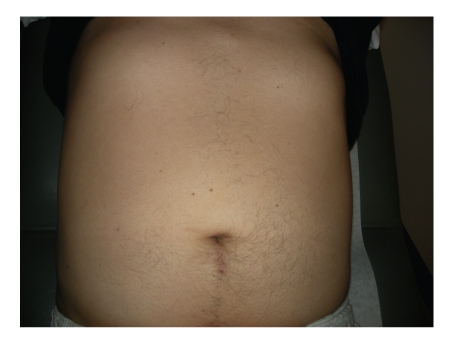
6 month postoperative image of surgical incision of LESS-PN.

Postoperatively the patients were started on Ketorolac and tramadol for pain. Narcotics were not given unless requested by the patient for breakthrough pain. Patients were given clear liquids on the same day of surgery and were then advanced as tolerated. CBC and SMA-7 were monitored postoperatively on daily basis. Patients were discharged on tramadol pro re- nata, and seen in the outpatient clinic within 1-2 weeks. Followup exam (Figure 4), laboratory and QoL determination, and imaging studies were obtained as per protocol depending on tumor pathology.

### 2.6. Data Collection and Analysis

Data were prospectively collected from the consent at the first clinic visit. Demographic factors [age, race, sex, BMI (Kg/m^2^), disease characteristics (tumor size, clinical and pathological stage, tumor pathology) perioperative variables [Operative time (minutes), EBL, incision size (cm), margin status, narcotic requirement (if any), time to advancement to regular diet (days) and length of hospital stay (days), and complications], quality of life- (QoL-) related outcomes [visual analog pain (VAP) score at discharge and 6 months postoperatively; time to return to normal activities (days), and SF-36 health survey (SF-36 V. 2.0; Medical Outcomes Trust, Inc., Boston, MA)], and disease outcomes (overall- and disease-specific survival) were recorded and analyzed for this initial cohort. Data was analyzed between the two groups (LESS-RN and LESS-PN) utilizing Student's *t*-test and Fisher's exact test, and for QoL and pain scores utilizing single-factor ANOVA with *P* < .05 is considered significant.

## 3. Results

Demographics, tumor characteristics, perioperative variables, and outcomes and complications for the LESS-RN and LESS-PN groups are summarized in the table. Mean age for the entire cohort was 58.7 ± 11.0 years (58.8 ± 5.9 years for LESS-PN, 58.7 ± 13.8 years for LESS-RN, *P* = .991) mean BMI was 27.0 ± 4.6 Kg/m^2^. Median followup was 9.8 months (range 8–12 months). Six lesions were in the left kidney and four were in the right; four tumors were in the mid pole, 3 in the upper pole, and 3 in the lower pole of the kidney. Two tumors had renal vein thrombi, one of which also had metastatic disease. Median tumor size on final histopathologic analysis for the LESS-RN group was 5.0 (range, 2.3–9) cm, and for the LESS-PN was 1.7 (range, 1.4–2) cm (*P* = .045).

LESS-RN was successfully performed on all 6 patients without conversion to multi-site laparoscopic or open surgery; two of these included successful enbloc resection of the renal vein thrombus, and one of which was also a cytoreductive nephrectomy. LESS-PN was successfully performed on 3 of four attempts with one conversion to open PN due to failure to progress, due to the patient's prior history of prior intraabdominal surgery and radiation. No significant differences were noted between the LESS-RN and LESS-PN groups with respect to perioperative variables: Mean operative time (LESS-RN 150.8 ± 27.6 versus LESS-PN 177 ± 24.1 minutes, *P* = .162), mean estimated blood loss (LESS-RN 133.3 ± 75.3 versus LESS-PN 112.5 ± 75.0 mL, *P* = .679), and mean incision size (LESS-RN 4.6 ± 0.5 versus LESS-PN 4.1 ± 0.3 cm, *P* = .143). The median hospital stay was significantly longer in LESS-PN (80 hours; range 73–88) compared to LESS-RN (52 hours; range 29–86, *P* = .048). Median warm ischemia time (for 3 tumors performed with ischemic technique) was 24 minutes (range 19–26). 

Histology revealed RCC in the majority of resected lesions, 5/6 (83%) LESS-RN and 2/4 (50%) LESS-PN. Of the seven patients with RCC, clear cell was the most common tumor (71%), one patient had a cystic RCC (14.3%) and one had a chromophobe RCC (14.3%). Three benign lesions were resected, two lipid poor angiomyolipomas and one oncocytoma. All patients had negative resection margins. 6-month postoperatively patients who underwent LESS-PN had excellent preservation of overall renal function with mean preoperative/postoperative values for serum creatinine (0.7 ± 0.1 versus 0.8 ± 0.2 mg/dL, *P* = .194) and estimated Glomerular Filtration Rate (99.3 ± 10.9 versus 86.8 ± 29.2 mL/min/1.73 m^2^, *P* = .419). 

One half the patients (overall and in each group) did not require further opiate supplementation of their postoperative analgesics. Mean VAP score at discharge, 1 month postoperative, 3 months postoperative, and 6 months postoperative was: 1.3 ± 1.3, 0.9 ± 0.7, 0.5 ± 0.9, and 0.4 ± 0.9, resp., *P* = .204). Mean preoperative, 1 month postoperative, 3 month postoperative, and 6 month postoperative SF-36 QoL Score was: 73.8 ± 10.4, 67.7 ± 10.8, 74.4 ± 10.4, and 77.1 ± 8.9, resp., *P* = .222). Mean time to return to normal nonstrenuous activity was 6.6 ± 2.9 days (median 6, range 3–12).

No patients required blood transfusions. One patient (10%) had a complication, a pneumothorax which was treated with tube thoracostomy decompression with resolution and tube removal within 23 hours. All patients are currently alive, and there has not been any evidence of radiographic progression in any of the patients with RCC.

## 4. Discussion

LESS is an emerging surgical technique which may promise further reductions in morbidity and improved cosmesis for patients. Emerging data on LESS has been reported in general surgical, gynecologic, and urologic procedures [17–22]. LESS for renal surgery was first reported in 2007 and since then a handful of authors have described variations of their technique in order to perform both RN and more recently PN for a variety of indications [10, 11, 23–25]. This report represents the first report in the literature of LESS-RN with renal vein thrombectomy and cytoreductive nephrectomy and the second group to report LESS-PN in adults. As such, this report further corroborates (partial nephrectomy) and demonstrates (renal vein thrombectomy) that increasingly complex procedures can be safely performed with the LESS platform. 

In order for LESS to become a viable alternative to traditional multi-site laparoscopy, it must first be proven to be feasible, safe, and reproducible and with equivalent outcomes. While there are few reported cases of LESS procedures, the existing data has demonstrated impressive initial outcomes comparable to traditional laparoscopy [26]. This is consistent with our experience; nine of ten cases were completed without the need for conversion to open or traditional laparoscopic technique, OR times to complete these were (mean total cohort) 161 minutes, EBL was 125 mL and no patients required blood transfusions. This is comparable to existing large multi-site series which demonstrate means of 105–201 minutes OR time, 172–300 cc EBL and 4.5% transfusion rates [4, 5, 8]. All patients had negative margins, and patients who underwent LESS-PN had mean WIT under 30 minutes, without significant changes in creatinine and eGFR, consistent with excellent short-term renal preservation. While the numbers are small, this is certainly also comparable to a large multi-institutional series of multi-site LPN which demonstrated a mean warm ischemia time of 30.7 minutes, and preoperative and postoperative creatinine of 1.01 and 1.18 mg/dL, respectively [8]. Complications rate was reasonable with only one patent having a significant complication despite the complexity and novelty of these procedures. As surgeons, OR staff, and technologies improve, we only anticipate these outcomes to improve further.

Once LESS has overcome the initial threshold and found to be comparable to the existing laparoscopic standard, it must offer a significant advantage for surgeons and patients to invest in this emerging technique. LESS allows RN and PN to be performed with fewer incisions as compared with traditional laparoscopic technique. The average incision size in this series was 4.42 cm with no need for any additional incisions. In addition to excellent postoperative cosmesis (Figure 4) afforded by substitution of multiple trocar sites in traditional laparoscopy by often almost imperceptible scars in the umbilical region, significant QoL benefits may be attained by minimization of abdominal incision. Prior work has demonstrated that decreasing incision size or specimen morcellation may decrease postoperative discomfort [6, 27]. However, while morcellation of renal masses may allow for reduction of incision size, it also results in distortion of renal architecture which may compromise accurate staging and grading of RCC [28]. Thus all LESS procedures were performed adhering to fundamental oncologic principles and tumors were extracted using intact specimen entrapment bags to prevent tumor seeding to the single incision site [29].

Consolidation of working trocars and the extraction incision into one site may result in reduced incisional morbidity as evidenced by limited need for narcotic medications in this series (Table 1). More than half of the patients in this cohort did not require any narcotic supplementation and of those that did, most did so for less than 24 hours postoperatively. This may in part be due to our pathway which places patients on Ketorolac immediately post operatively [30, 31]. The resultant benefits of this are translated into preserved quality of life as evidenced by low discharge mean visual analog pain score (1.3), returning to normal nonstrenuous activity in less than one week, the lack of significant difference between preoperative and postoperative SF-36 scores (*P* = .222).

Since LESS procedures are relatively new and in evolution, many techniques have been described but no widely accepted standard exists. LESS has gained recent interest, and this has lead surgeons to use traditional tools in unique ways as well as encouraging industry to develop a variety of novel platforms and innovative instruments to ease the learning curve and facilitate these procedures. The gelport laparoscopic system (Applied Medical, Rancho Santa Margarita, CA) [11] is a laparoscopic hand port which allows introduction of multiple trocars while maintaining an airtight seal. Specialty designed single-port laparoscopic systems such as the Uni-X (Pnavel Systems, Morganville, NJ) [32], R-port (Advanced Surgical Concepts, Dublin, Ireland) [10, 23, 24], and the SILS port (Covidien, Mansfield, MA) are essentially multiple fixed trocar ports that are inserted through modified Hasson technique. We believe that our technique of using multiple traditional and low-profile trocars placed through a single incision offers some significant advantages. Both the gelport and the specially designed multisite trocars add additional cost to the procedure. Furthermore, specially designed multisite ports have fixed positions which limit separation of the trocars and prevent the use of additional trocars. In 3 cases we added a 4th trocar in the most caudal aspect of the incision, a 12 mm trocar allowed for the insertion of an Endo Paddle retract (Covidien), a retractor used for bowel retraction used for 2 patients with renal vein thrombi and one large upper pole renal mass. While some have suggested that the drawback of our method of LESS is the “swiss cheese” defect and weakening of the fascia, but we have not found this to be the case [33]. 

Triangulation is the primary underlying technical principle in laparoscopy and the greatest hurdle to overcome in LESS. Proximity of the working ports through the single incisions limits achievable separation which is necessary for triangulation. A variety of novel trocars, ports, and instruments have been developed specifically for or adapted for LESS. While we enthusiastically encourage the development of such products and believe that these will improve the technical feasibility of these surgeries our initial experience has been performed without the use of any such specialized tools. We believe that the benefits of using conventional laparoscopic trocars and instruments are primarily two fold: (1) surgeon familiarity and comfort, (2) cost savings [minimizing the use of flexible/articulating instruments which are disposable]. Given that our OR times, and other outcomes are consistent with those of large published series of multiport RN and LPN [4, 5, 8], we feel that our approach of utilizing conventional laparoscopic instruments facilitates surgeon comfort and safe adoption of the LESS platform with excellent results.

We believe that our utilization of extra-long laparoscopic instruments and cameras creates a zone of extracorporeal triangulation which, when applied through a peri-umbilical incision which is close to the kidney, creates sufficient working freedom and attenuates clashing. Furthermore, our utilization of staggered trocars of lengths and a right-angled camera further minimizes instrument clashing and allowed greater angulation of laparoscopic handles. Thus we have demonstrated the LESS renal surgery can be performed using essentially the same tools that might be used to perform traditional multisite laparoscopy. Articulating instruments, trocars that allow the insertion of bent instruments and flexible laparoscopes all may provide further methods of overcoming these challenges and are in the process of being further evaluated by our group.

Despite advances in LESS considerable challenges remain. The upper posterior pole of the kidney is the most difficult portion of the renal dissection. Even with the use of bariatric laparoscopic instruments this region is difficult to reach because of the greater working distance from the umbilicus, and “turning the corner” or getting over the upper pole to the posterior aspect of the kidney can be demanding with rigid instruments that do not articulate. Additionally retraction of the bowel and liver without multisite laparoscopy is challenging. While future advances in laparoscopes, trocars, and instruments may overcome these technical considerations, novices to LESS renal surgery may consider extra caution in attempting these procedures in patients with greater BMIs and patients with bulky upper pole posterior lesions. Particular consideration must be employed when attempting LESS-PN; while intracorporeal suturing is feasible for obtaining hemostasis and closure of the renal collecting system and parenchyma, tumors that may not be easily accessible from the umbilicus due to distance (such as posterior, upper pole lesions) may present further difficulties and present onerous limitation on being able to deploy laparoscopic needle drivers at a sufficient angle. Indeed, development and refinement of robotically-assisted LESS may allow a greater degree of freedom and surmount such difficulties.

Increased detection of small renal masses has required urologist to gain familiarity with procedures that ensure adequate oncologic control while preserving renal function [34]. LESS-PN allows for extraction of the enhancing renal lesion, definitive histologic confirmation with excellent preservation of renal function in this series. In a recent publication, Kaouk and Goel utilized a nonischemic technique to perform LESS-PN. After PN these authors achieved hemostasis using ABC, Surgicel and a variety of surgical adhesives, however due to inability to achieve adequate hemostasis in one case they had to convert to multiport laparoscopy [25]. We attempted a nonischemic technique in one case, utilizing the Habib 4X laparoscopic radiofrequency resection device, which easily fits through the 12 mm laparoscopic port. This device allows excision of the renal mass while maintaining hemostasis by ablating normal renal parenchyma and creating an avascular plane around the tumor allowing excision of the mass with minimal blood loss and preserving histologic integrity of the specimen [16]. 

Despite the novelty of these procedures we rapidly adopted an excellent comfort level for performing complex LESS-RN. Two patients had renal tumors greater then 7 cm, both with renal vein thrombi. One of these patients had widely metastatic disease and elected to undergo cytoreductive LESS-RN. Traditional laparoscopic cytoreductive nephrectomy has been demonstrated to have favorable morbidity when compared with open technique and thus we performed to our knowledge the first reported LESS cytoreductive nephrectomy [35]. The patient did well and was able to resume targeted biologic therapy on postoperative day 14.

The limited number of procedures and the lack of a direct comparison to traditional multi-site LRN and LPN limit our findings. However, this preliminary prospective series demonstrates that LESS-RN, renal vein thrombectomy, and PN is safe and technically feasible method for performing complex renal surgery while maintaining strict adherence to oncologic principles, with excellent preservation of quality of life, low discharge pain scores, and cosmetic benefit. Our encouraging pilot results have led to a prospective comparison between LESS and multiport laparoscopy, which we hope will delineate what, if any specific advantages, may lie with the LESS approach.

## Figures and Tables

**Table 1:** Demographics, Tumor Characteristics, Perioperative Variables, and Outcomes.

	LESS-RN (*n* = 6)	LESS-PN (*n* = 4)	*P* value
*Demographics*			

Age	58.7	58.8	.991
Sex (Male/Female)	2/4	1/3	.806
BMI (Kg/m2)	24.9	30.1	.073

*Tumor size/location*			

Tumor Size (cm)	5.3	1.7	.045
Tumor Location			
Upper/Mid/Lower Pole	2/2/2	1/2/1	
Renal Vein Thrombus	2	0	
*Perioperative Variables*			

Operative Time (minutes)	150.8	177.0	.162
Estimated Blood Loss (mL)	112.5	133.3	.679
Warm Ischemia Time (minutes)	n/a	22	
Incision size (cm)	4.6	4.1	.143
Narcotic Requirement (yes/no)	3/3	2/2	1.000
Hospital Stay (hours)	54.4	80.3	.048

*Outcomes/Complications*			

Conversion to open	0/6 (0%)	1/4 (25%)	.241
Negative Margins	6	4	1.000
Malignant Histology	5	2	.312
Blood Transfusion	0	0	1.000
*Complications*	1	0	.241
Overall Survival	6	4	1.000
